# The yolk sac as the main organ in the early stages of animal embryonic development

**DOI:** 10.3389/fphys.2023.1185286

**Published:** 2023-05-22

**Authors:** Irina V. Kuzmina

**Affiliations:** Laboratory Physiology of Motivations, Federal State Budget Scientific Institution “P. K. Anokhin Research Institute of Normal Physiology”, Моscow, Russia

**Keywords:** yolk sac, embryo, birds, mammals, fish

## 1 Introduction

The yolk sac is the first of the extra-uterine organs to emerge in the course of evolution. In marsupials, the yolk sac, devoid of yolk, persists throughout pregnancy, and in placental mammals, it completes its development at the early stages of embryogenesis (Single ungulates and cloven-hoofed, and humans). In some species, the true yolk placenta exists until the end of pregnancy (rabbits and rodents). In reptiles, the yolk sac is associated with an intermediate villous membrane, the chorion (an organ that is closely related to the maternal organism), which forms a placental connection with the uterus while performing its trophic function. It is also known that during evolution, the allantois loses its trophic and respiratory functions but retains its leading role as a reservoir of metabolic products. In some cases, it disappears altogether (marsupial marten) or forms a dwarf allantois placenta (marsupial badger) (Krause and Cutts, 1984). The fate of the allantois directly depends on the competitive interaction with the yolk sac because, in the course of ontogenesis, it is the first to approach the serous membrane. In the course of these interactions, redistribution of fluid between tissues of the fetus and extra-germinal organs occurs in accordance with the requirements imposed on the forming organism by the external environment (Sklyanov et al., 2005). Nevertheless, as a manifestation of recapitulation, the yolk sac persists and then establishes new, compared to those in reptiles and birds, relations between the extra-germinal organs to implement their functions.

## 2 Opinion

### 2.1 Utilization of the yolk sac in birds

During incubation, the growth and development of the avian embryo depend on nutrients contained directly in the egg (Yadgary and Uni, 2012). For this, the avian embryo can solely rely on nutrients from the yolk, protein, and shell. The yolk sac membrane performs numerous metabolic functions and is the first line of defense against pathogens in the yolk. In terms of functions performed, this tissue acts as bone marrow for the synthesis of blood cells, the intestine for digestion and the transportation of nutrients, the liver for the synthesis of plasma carrier proteins and carbohydrate exchange, the thyroid for metabolic regulation, and the immune system for the transmission of antibodies from the hen. The rate of utilization of the contents of the residual yolk sac is related to the metabolic intensity of the embryo as well as to its size and composition at hatching (Nangsuay et al., 2013). Due to the fact that the chick receives mainly nutrients in the form of proteins and carbohydrates from feed at a day old, it can still use the lipids stored in the remnants of the yolk sac, even when its membrane is destroyed. In addition to this, any disruption in the absorption of yolk sac contents can lead to a lack of essential nutrients and maternal antibodies, leading to mortality and poor poultry quality (Wong and Uni, 2020). It can be assumed that the utilization of the residual yolk sac during incubation and in the early post-embryonic period can affect the quality and development of chicks in the first week of rearing, as well as the development and productivity of poultry in age-related ontogeny. Podobed (2013) also states that the faster the chick gets rid of the remnants of the yolk sac, the faster it develops the ability to maximize the digestion of nutrients from the feed. Dissolution of the yolk sac in poultry in the first days of post-embryogenesis goes in parallel with the formation of their digestive system. Previously, our research results showed that the contents of the yolk sac of daily broiler chickens have high proteolytic activity. By 3 days of age, trypsin activity decreased by almost half, whereas lipolytic activity increased compared with the day-old chickens. We concluded that in the post-embryonic period of the chick, the yolk sac has no significant effect on the development of the pancreas. However, there was a stable inverse relationship: the smaller the weight of the yolk sac, the greater the weight of the duodenum (Vertiprakhov, 2022). This suggests that chickens fed immediately use more yolk than chickens fed with a delay, which may be due to higher intestinal enzymatic activity, which is probably due to peristaltic movements (Noy et al., 1996). However, the study of morphophysiological features of the yolk sac remains an interesting area of research despite the extensive information available in the literature.

### 2.2 Use of the yolk sac in mammals

The most primitive mammals are egg-laying mammals. Their embryogenesis is similar to that of birds. In marsupials, eggs contain a small amount of yolk, but the embryo is born underdeveloped and its further development proceeds in the maternal sac, where the mother’s mammary gland nipple is connected to the baby’s esophagus. For higher mammals, intrauterine development and nutrition of the embryo at the expense of the mother’s body is characteristic, which is reflected in embryogenesis. Eggs are almost completely secondary to the loss of the yolk. They develop in the follicles of the ovary. After ovulation, they enter the oviduct. One of the peculiarities of mammalian development is the very early separation of the germ from the non-germ. Simultaneously with the formation of the embryonic body, one of the fetal membranes—the yolk sac—develops. The yolk sac is formed from the extra-germinal entoderm and the visceral sheet of the mesoderm. It contains a protein fluid. The wall of the yolk sac forms blood vessels. This shell performs the functions of hematopoiesis and trophic function (Bunkova, 2020). As a reservoir of yolk, the yolk sac provides its storage, cleavage, and absorption of formed monomers in a well-developed vascular network (Dubinina and Sklyanov, 2011). Placental mammalian and human oocytes are practically devoid of yolk. Nevertheless, the yolk sac in them is not only formed but also preserved until birth. This extra-natal organ (unlike that of rodents studied in laboratory experiments) belongs to the “free”-type, as it has no direct contact with tissues of the maternal organism (Freyer and Renfree, 2009). Located in the cavity of the exocelome and washed by its contents, the yolk sac appears to be able to absorb the substances it contains through its outer surface. The close connection of the exocelomic epithelium with the blood vessels also suggests the possible participation of the yolk sac tissues in providing the embryo (fetus) with nutrients through the yolk circle of vessels. Thus, the period of active functioning of the exocelomic epithelium of the yolk sac of mammals and humans is limited to the first trimester of pregnancy. This seems to be primarily due to the rapid growth of the fetus, leading to a decrease in the exocelome cavity and an increase in compression. Another possible explanation for what is happening may be a gradual change in the type of fetal nutrition. Anyway, the histogenesis and functioning of the exocelomic epithelium of the mammalian yolk sac with its subsequent involution is another striking example of the accelerated differentiation and specialization of tissues comprising the vertebrate extra-uterine organs.

### 2.3 Use of the yolk sac in fish

During gastrulation in fish, the first extra-germinal organ, the yolk sac, is formed, and it provides for the further development of the embryo. The epidermal layer of the yolk sac produces cells that break down the yolk into constituent elements that go toward feeding the embryo. Blood vessels appear in the mesodermal layer, through which nutrients flow to the embryo. The outer ectodermal layer of yolk sac cells performs gas exchange. The yolk sac is connected to the midgut cavity by the yolk stalk. As the nutrient material of the yolk is consumed, the sac retracts into the body of the embryo, the entoderm becomes part of the intestinal wall, the ectoderm becomes part of the skin, and the vascular system of the yolk sac is reduced. Thus, the yolk sac is a provisory (temporary) organ appearing in the evolution of vertebrates (Kokorina, 2010). The yolk sac in fish embryos should be considered, first of all, from the point of view of the necessity of this organ for food from the early stage of growth and development to the transition to mixed feeding of fish fry. The energy function of the yolk sac in fish can be considered in terms of a closed embryo–yolk system. Following the law of conservation of energy, in the yolk sac in fish embryos, there are additional irretrievable losses of nutrients for the formation of embryonic respiratory organs and mechanisms (Buslov and Sergeeva, 2013).

## 3 Discussion

The yolk sac is a feeding and breathing organ in the embryos of cephalopod mollusks, cartilaginous and bony fish, reptiles, birds, mammals, and humans. It appears at the early stages of embryonic development usually by fouling of the yolk with the entoderm and visceral sheet of the lateral plates and is an expanded outgrowth of the middle intestine, the cavity which, in most animals (except higher mammals and humans), is filled with unshattered yolk. Blood cells and blood vessels are formed in the wall of the gut, providing for the transfer of nutrients to the embryo and its respiration. With the development of the embryo, the size of the yolk sac and its cavity decrease, and it gradually retracts into the body cavity and is either resorbed or rejected. In this regard, this organ can be considered one of the main organs due to its hyperfunctionality.
